# Characteristics of the autism spectrum disorder gastrointestinal and related behaviors inventory in children

**DOI:** 10.1002/aur.2707

**Published:** 2022-03-18

**Authors:** Calliope Holingue, Luther G. Kalb, Rashelle Musci, Colleen Lukens, Li‐Ching Lee, Jamie Kaczaniuk, Michelle Landrum, Timothy Buie, M. Daniele Fallin

**Affiliations:** ^1^ Department of Mental Health Johns Hopkins Bloomberg School of Public Health Baltimore Maryland USA; ^2^ Wendy Klag Center for Autism and Developmental Disabilities Johns Hopkins Bloomberg School of Public Health Baltimore Maryland USA; ^3^ Center for Autism and Related Disorders Kennedy Krieger Institute Baltimore Maryland USA; ^4^ Department of Child and Adolescent Psychiatry and Behavioral Sciences at The Children's Hospital of Philadelphia Philadelphia Pennsylvania USA; ^5^ Department of Epidemiology Johns Hopkins Bloomberg School of Public Health Baltimore Maryland USA; ^6^ Division of Gastroenterology, Hepatology and Nutrition Harvard Medical School Boston Massachusetts USA

**Keywords:** autism, co‐morbid conditions, psychometrics, questionnaire

## Abstract

Gastrointestinal (GI) symptoms are one of the prevalent co‐occurring issues in autism spectrum disorder (ASD), though the range of symptom frequency estimates varies dramatically across studies, which can limit the further research of GI issues in ASD as well as potential treatment strategies. The wide range of prevalence estimates is partly due to the lack of standardized, validated measures of GI symptoms among people with ASD. The goal of this study was to (1) develop a measure, which included non‐verbal and mealtime behaviors, to assess for GI symptoms and (2) evaluate its psychometric characteristics. This was accomplished by drawing on two existing tools, Autism Treatment Network Gastrointestinal Inventory and the Brief Autism Mealtime Behavior Inventory, and deriving new items, to create the “ASD Gastrointestinal and Related Behaviors Inventory” (ASD‐GIRBI). The ASD‐GIRBI was piloted in an online registry of families with a child with ASD. A psychometric analysis was carried out in a sample of 334 children aged 6–17 years with ASD, resulting in a 36‐item tool. The Cronbach's alpha for the overall scale was 0.88. Exploratory factor analysis identified a seven‐factor model (1. Bowel movement pain; 2. Aggressive or disruptive during mealtimes; 3. Particular with foods; 4. Abdominal pain and upset stomach; 5. Refusing food; 6. Constipation and encopresis; 7. Motor or other behaviors). Following validation in an independent sample with clinical evaluation of GI symptoms, this tool will be helpful for both research and clinical purposes.

## Lay Summary

Gastrointestinal (GI) symptoms are common in children with autism spectrum disorder (ASD), yet assessing these symptoms remains challenging. This study developed a parent‐report, 36‐item questionnaire to measure GI symptoms in children with ASD. The tool included questions about food, mealtimes, GI symptoms, bowel habits, and behavior. This questionnaire may help researchers and clinicians assess GI symptoms among children with ASD.

## INTRODUCTION

Gastrointestinal (GI) disorders are one of the most prevalent medical conditions in autism spectrum disorder (ASD), along with sleep disorders and seizures/epilepsy (Bauman, 2010; Mannion et al., 2013). The most common GI symptoms found in ASD are chronic constipation, diarrhea, or alternating constipation/diarrhea, abdominal pain, and acid reflux, though other symptoms are elevated as well (Buie et al., 2010; Margolis et al., 2019). GI symptoms are distressing because of the pain, discomfort, and functional limitations they produce and their effect on mental and physical health. Individuals with ASD with co‐occurring GI symptoms are more likely to have sleep disruptions (Klukowski et al., 2015; Maenner et al., 2012), aggressive, irritable, externalizing, or self‐injurious behaviors (Christensen et al., 2009; Fulceri et al., 2016; Maenner et al., 2012; Mazefsky et al., 2014; Mazurek, Kanne, & Wodka, 2013; Mazurek, Vasa, et al., 2013), anxiety and mood problems (Fulceri et al., 2016; Mazefsky et al., 2014; Mazurek, Kanne, & Wodka, 2013; Mazurek, Vasa, et al., 2013), sensory sensitivities/over‐responsiveness (Mazurek, Kanne, & Wodka, 2013; Mazurek, Vasa, et al., 2013), toileting problems such as soiling (Radford & Anderson, 2003), food sensitivities and eating issues (Coury et al., 2012; Klukowski et al., 2015; Kral et al., 2013; Vissoker et al., 2015), and other psychopathology and somatic issues (Fulceri et al., 2016; Mannion & Leader, 2014; Peters et al., 2014).

In Leo Kanner's 1943 paper first describing autism, he wrote that six of the 11 children with autism “presented severe feeding difficulty from the beginning of life” (Kanner, 1968). Decades of studies since have also found that individuals with ASD tend to have more GI symptoms than their typically developing counterparts (Buie et al., 2010; Chaidez et al., 2014; McElhanon et al., 2014). However, the range of symptom frequency estimates has varied dramatically across ASD studies, for example, from 4%–97% for any GI symptom (median 47%), 2%–76% for diarrhea (median 13%), 4%–45% (median 22%) for constipation, and 1%–22% (median 6%) for nausea/vomiting (Holingue et al., 2018). This lack of precision limits the ability to carry out rigorous epidemiologic and therapeutic research on the overlap between GI symptoms and ASD. The imprecision of symptom frequency estimates is in part due to the heterogeneity of ASD, and notably also due to the lack of standardized and validated tools evaluated in ASD populations (Buie et al., 2010; Holingue et al., 2018).

One major limitation is that people with ASD, particularly children, may have difficulties self‐reporting or describing medical symptoms, including GI symptoms and pain. For example, children with ASD may not spontaneously approach their parents complaining of “tummy pain” or respond affirmatively when asked if their “belly hurts” despite experiencing GI pain. Even children with ASD and fluent speech may not communicate GI distress to their parent/caregiver in a typical way. Therefore, parents of children with ASD often rely on non‐verbal behaviors (e.g., sleep difficulties, irritability, aggression) and bodily signs (e.g., abdominal swelling, gas, diarrhea) to recognize when their child is experiencing GI symptoms. Questionnaires often used in typically developing children do not usually contain a sufficient range of non‐verbal behaviors to capture GI symptoms in children with ASD, such as facial grimacing, unusual posturing, self‐injurious behavior (Buie et al., 2010).

Further, GI questionnaires with items about mealtimes typically do not include the restrictive, repetitive, or sensory types of mealtime behaviors common in ASD (Lukens & Linscheid, 2008). For example, the PedsQL asks about a child's inability to eat or drink food they want to eat but does not ask about food preferences, refusal to eat food, or other behaviors around a mealtime (e.g., spitting out food, turning away from food, disruptive during mealtimes) (Varni 2022). The inclusion of these mealtime items is vital for several reasons. First, it is possible that being particular about food or refusing food could be due to past adverse GI reactions to a specific food or type of food (perhaps misattributed to sensory aversion). Second, a very restrictive diet can contribute to GI issues, such as constipation due to inadequate fiber intake (Harris et al., 2021). Next, previous literature has found that eating/feeding issues are associated with GI dysfunction in the pediatric ASD population (Ming et al., 2008; Vissoker et al., 2015). For example, Fields et al. found high frequencies of feeding problems among all children with gastroesophageal reflux in their study (Field et al., 2003). Given the difficulties of assessing reflux, the inclusion of dietary or mealtime items may be helpful for recognition.

There are ASD‐specific GI questionnaires, but they also have limitations, including omission of mealtime or eating behavior problems, or behavioral symptoms that may be indicators of GI distress. To date, only one GI questionnaire or screener, the Autism Speaks Autism Treatment Network GI Signs and Symptoms Inventory‐17 (AS‐ATN GI Signs and Symptoms Inventory‐17), published in 2019, has reported psychometric properties among those with ASD (Margolis et al., 2019). This tool, relative to others, has the advantage of including GI‐motoric items which may be particularly helpful in identifying GI distress in a non‐ or minimally‐verbal child with ASD. However, this tool had a limited number of items having to do with mealtime or dietary preferences/behaviors, which might be reflective of or associated with GI symptoms (Margolis et al., 2019).

Given the lack of ASD‐specific GI measures, and the known burden of disease in children with ASD, the goal of this study was to (1) develop a parent‐report measure, which included non‐verbal and mealtime behaviors, to assess GI symptoms and (2) evaluate its psychometric characteristics. This was accomplished by drawing on two existing tools, the ATN‐GI Inventory and the Brief Autism Mealtime Behavior Inventory (BAMBI), as well as deriving new items, to create the “ASD Gastrointestinal and Related Behaviors Inventory” (ASD‐GIRBI).

## METHODS

### Preliminary scale development

To develop constructs and the item pool for the questionnaire, a review of the literature on approaches to assessing GI symptoms in epidemiologic studies of ASD (Holingue et al., 2018) was carried out, as well as expert discussion and information gathered from qualitative interviews with individuals with ASD and their parents. First, with permission from the authors, items were extracted from the ATN‐GI (Network, 2005) and the BAMBI (DeMand et al., 2015; Hendy et al., 2013; Lukens & Linscheid, 2008). The ATN‐GI Inventory was developed by pediatric gastroenterologists from the Autism Speaks‐Autism Treatment Network and was designed to assess for functional constipation, functional diarrhea, and gastroesophageal reflux disease (GERD) (Margolis et al., 2019). This measure was reduced into the aforementioned AS‐ATN GI Signs and Symptoms Inventory‐17, a 17‐item screener which consists of four dimensions or factors: Retentive, Expulsive, Gas, and Motoric. The tool had a sensitivity of 84%, specificity of 43%, and a positive predictive value of 67% for identifying children with one more of the following GI disorders: functional constipation, functional diarrhea, or GERD. The advantage of this tool, relative to others, is its inclusion of GI‐motoric items such as “In the last three months, did your child appear to feel pain when having a BM [bowel movement]?” or “In the last three months, did your child push his abdomen with his/her hands or your hands, push his/her abdomen against or lean forward over furniture?”

The BAMBI, an 18‐item caregiver‐report questionnaire, was designed to evaluate mealtime behaviors in children with ASD and has been shown to have good internal consistency, high test–retest reliability, and strong criterion‐related validity (Lukens & Linscheid, 2008). The BAMBI is comprised of three factors: limited variety, food refusal, and features of autism. The limited variety factor relates to restricted food preferences, such as the child being willing to try new foods or preferring the same foods at each meal. The food refusal factor relates to rejection of food, such as the child closing their mouth tightly when food is presented or expelling food they have eaten. Lastly, the features of autism factor relates to behavioral characteristics of, or associated with, autism, such as inattention, self‐injurious behavior, or rigid behavior patterns (Lukens & Linscheid, 2008).

Next, de novo items were added to the pool, based on a review of the literature, qualitative interviews of children with ASD and their parents (described below), and the combined expertise of this coauthor team on ASD, gastroenterology, nutrition, psychometrics, epidemiology, and public health. The decision to add de novo items to the tool was informed by both an awareness that previous tools were missing important items and the appreciation that stakeholder input is valuable.

Before, during, and after the development of our initial GI inventory, qualitative interviews were held with parents of children with ASD and GI symptoms to determine whether constructs or items were missing from the inventory and to ensure content validity. Individuals were eligible to participate if they were the parent or caregiver of a child with ASD who had a history of GI symptoms between 3 and 18 years. In some cases, their children would join the interview. Adults with ASD who had GI symptoms as children were also eligible. One such adult participated. The methodology and findings of these qualitative interviews are described in depth elsewhere (Holingue et al., 2021 *Autism*). In brief, however, 12 qualitative interviews each took 30–45 min. They were held either in person in a private location such as the participant's home or through a video/audio conversation using the Zoom Video Conferencing Platform. Participants were asked questions such as “What are the GI issues your child currently struggles with or has struggled with in the past? What are things you notice about your child when they are having GI symptoms/distress? What are some signs/behaviors that you see? What areas related to GI issues have affected your family or your child's functioning?” Participants were probed to expand on their experiences, provide examples, or clarify any remarks. This exploratory store drew on phenomenology to understand lived experiences and how parents identify the child is experiencing GI symptoms (Creswell et al., 2007). We used an inductive coding process to derive meanings, or themes, from the raw textual data (Hsieh & Shannon, 2005; Thomas, 2006). The current study's questionnaire included bodily signs (e.g., flatulence) and non‐verbal behaviors (e.g., unexplained irritability) that parents reported using to detect GI distress in their children in these interviews.

Lastly, two in‐person cognitive interviews were carried out in which parents completed a sample of items on the tool and were asked to provide feedback on the clarity of wording, the relevance of the item, what construct the item conjured for them, and whether the items were upsetting or insensitive. Revisions were made to the questionnaire based on these cognitive interviews.

### Participants and procedure

**FIGURE 1:**
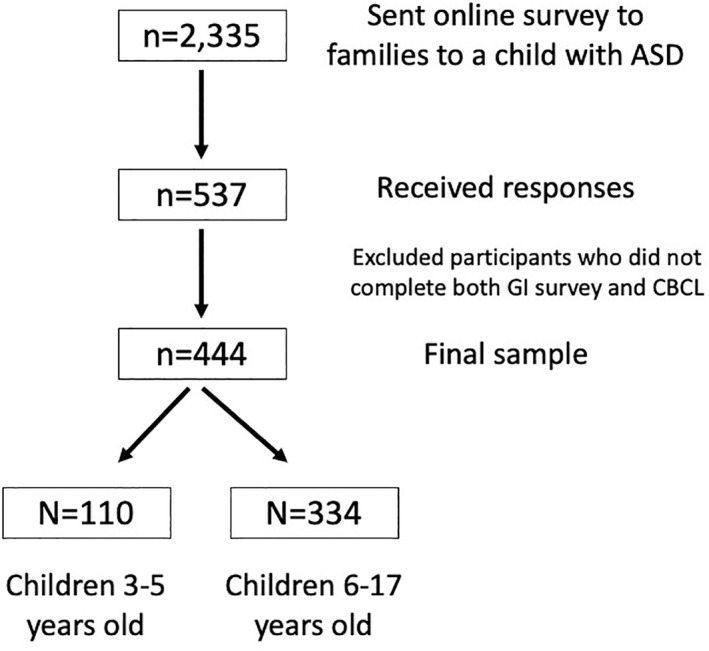
Flow chart of study participant recruitment

Participants were recruited from a registry of parents with a child with ASD at Kennedy Krieger Institute (KKI). This center combines research, clinical service, therapeutic day programs, and training programs for children with developmental disabilities and disorders of the brain, spinal cord, and musculoskeletal system. These parents had previously consented to be contacted for research purposes. Parents with a child with ASD between the ages of 3–18 years were eligible to participate in the study, regardless of the child's experience with GI symptoms. An invitation was sent to the 2335 eligible families (Figure 1). If parents were interested in participating in the study, they were provided a Qualtrics survey link, which included an online consent form and the study measures. All data collection happened electronically. The parent or primary caregiver was the informant for all children and young adults with ASD (ages 3–17 years). Of the 2335 eligible participants, 537 consented to complete the survey. Individuals who did not complete both the GI tool and child behavior checklist (CBCL) were excluded (*n* = 93), leaving 444 children (83%) (Figure 1).

### Measures

ASD diagnosis was determined prior to this study by a licensed medical provider (e.g., psychiatrist, neurodevelopmental pediatrician) or licensed psychologist (clinical or neuro) based on the diagnostic and statistical manual of mental disorders, version 4 or 5. Information from a previous administration of the autism diagnostic observation schedule‐2 (ADOS‐2) (Lord et al., 2012) was used to determine the diagnosis.

#### Child behavior checklist

Participants from the KKI research registry completed either the CBCL 1.5–5 or the CBCL 6–18, depending on their child's age. The CBCL is a reliable, valid questionnaire completed by the parent/caregiver who spends the most time with the child. The CBCL can be completed at home in 10–20 min. For each problem item, such as “disturbed by any change in routine,” parents are asked to rate how true each item is for their child is in the past 6 months (for ages 6–18 years) or past 2 months (for 1.5–5 years): not true, somewhat or sometimes true, or very true or often true (Achenbach & Rescorla, 2003; Achenbach & Ruffle, 2000; Pandolfi et al., 2009). Each syndrome domain can be scored in the normal range, in an area of concern but not considered clinical, or in the clinical range, based on scores from a national normative sample. A score was derived for each of the following CBCL domains: anxiety/depression, emotionally reactive, somatic complaints, withdrawn, attention problems, aggressive behavior, and sleep problems (Pandolfi et al., 2012).

#### GI questionnaire (ASD‐GIRBI)

The GI questionnaire consisted of 56 core items. This core set of questions included four sections, organized by type of symptom or behavior: (1) presence of 11 GI symptoms in the last 3 months, (2) five items on frequency of BMs and stool consistency (Bristol stool chart; Riegler and Esposito 2001) in addition to seven items on toileting behaviors, (3) 20 items on mealtime and dietary behaviors, and (4) 13 other behaviors (e.g., unexplained irritability, agitation, aggression, or screaming; chewing on shirts, eating non‐edible objects; pointing to stomach/tummy as if in pain). Outside of these core 56 items, parents were also asked to report physical/mental health diagnoses in the child (*n* = 14 items), medications their child was taking, how GI symptoms impacted their child's functioning, and their confidence in accurately assessing their child's pain level. Items on symptom duration and associations with BMs, eating, and weight were also included as sources of additional information. The complete questionnaire can be found in the Supplement.

### Analysis plan

A psychometric assessment of the ASD‐GIRBI was carried out by performing exploratory factor analysis (EFA), assessing the tool's reliability with Cronbach's alpha, and assessing convergent validity. The 56 core GI questionnaire items were used for psychometric assessment. Items were dichotomized as present rarely/never versus several times per month or more. Responses of “not sure” were labeled as missing. All data cleaning and analyses were performed in R Studio version 1.1.383 (R version 3.4.3). Because either of two CBCL versions was administered (ages 3–5 and 6–17 years) and only 110 individuals 3–5 participated, factor analysis and reliability and convergent validity assessments were only performed in the group 6–17 years of age.

EFA was performed to determine the factor structure of the GI questionnaire. The 5‐step procedure recommended by Costello and Osborne (Osborne et al., 2014) was followed. Items endorsed by <10% of individuals were dropped. The redundancy of items was assessed by calculating pairwise correlations between items. Next, items that decreased the scale's internal consistency (Cronbach's alpha) were dropped. This was done because if a scale's alpha increases when an item is removed, it indicates that the scale's reliability is higher with the omission of the item compared to that of the complete set.

The *Psych* package in R (Revelle, 2017) was used to perform parallel analysis of principal components using ordinary least squares to identify the minimum residuals to extract factors. Oblimin rotation (oblique) was used, allowing factors to be correlated with each other. Items that did not load onto a factor (score < 0.30) were dropped, after which the factor structure was evaluated using fit indices, including the root mean square error of approximation (RMSEA) and the Tucker‐Lewis index (TLI) (Bentler, 1990; Marsh et al., 1988). Factor scores were calculated by taking the sum of the number of items endorsed by the person for each factor. For example, if a factor consisted of seven items, individuals who endorsed all seven items would receive a factor score of seven, while those who did not endorse any of the items would receive a score of 0.

Convergent validity reflects evidence of similarity between theoretically and conceptually related constructs. This was assessed by estimating associations between factor scores and subscales on the CBCL, and parent‐report GI diagnoses (DeVellis, 2016). Associations with directions and magnitudes as expected suggest good convergent validity. CBCL subscales were included given the extant literature linking internalizing and externalizing symptoms with GI problems in children with ASD (Ferguson et al., 2019).

Lastly, the sensitivity, specificity, and area under the curve (AUC) of Receiver Operating Characteristic (ROC) Curves were calculated to assess which factor scores predicted self‐reported GI diagnoses. A cut‐off score of 1 on the ASD‐GIRBI was used for each factor score to maximize sensitivity.

## RESULTS

### Participant characteristics

As stated above, of the 2335 study invitations sent to KKI registry families, 537 participants consented to complete the survey. Individuals who did not complete both the GI tool, as well as the CBCL, were excluded (*n* = 93), leaving 444 children (Figure 1). The majority (75%) of these children were between 6 and 17 years old (Table 1). Over 90% of participants who completed the survey on behalf of their child were mothers and highly educated, with 89%–90% having some college/AA education or greater. Children were mostly male (83% in 3–5‐year‐olds, 78% in 6–17‐year‐olds). Just over half (52%–59% of participants were white, 18%–25% were Black or African‐American, 14%–16% Multiracial, 7%–8% Asian, and 7%–9% Hispanic/Latino). The most commonly reported medical diagnoses were allergies/asthma, GI disorders, and sleep disorders. The most common psychiatric/developmental disorders endorsed were sensory processing disorder, anxiety, panic or phobia disorder, ADD/ADHD, intellectual disability, and obsessive–compulsive disorder (Table 2).

**TABLE 1:** Demographic characteristics of study participants (% or mean [SD])

	Ages 3–5 years (*n* = 110)	Ages 6–17 years (*n* = 334)
Child age (years)	4.3 (0.75)	9.5 (3.09)
Respondent relationship to child		
Mother	93%	92%
Respondent education level		
High school graduate/Tests of General Educational Development (GED) or below	12%	11%
Some college/Associates Degree (AA) education	32%	19%
College/AA degree	25%	32%
Graduate education	32%	39%
Child biological sex		
Female	22%	25%
Male	78%	75%
Child gender identity		
Female	17%	21%
Male	83%	78%
Non‐binary, gender‐queer, gender‐fluid, or transgender	0%	1%
Child race/ethnicity		
White	52%	59%
Black/African‐American	25%	18%
Multiracial	14%	16%
Asian	8%	7%
Hispanic/Latino	7%	9%

**TABLE 2:** Parent‐reported psychiatric and medical diagnoses of study participants

	Ages 3–5 years (*n* = 110)	Ages 6–17 years (*n* = 334)
Confidence in ability to assess your child's GI pain		
Not confident at all	13%	12%
Slightly confident	35%	23%
Fairly confident	42%	46%
Completely confident	11%	18%
Psychiatric and medical diagnoses		
Medical conditions		
Allergies or asthma	27%	39%
Any gastrointestinal disorder	19%	20%
Sleep disorder	8%	16%
Seizure/epilepsy disorder	2%	7%
Autoimmune disorder	2%	3%
Psychiatric/neurodevelopmental conditions		
Attention‐deficit/hyperactivity disorder (ADHD)	12%	55%
Sensory processing disorder	50%	49%
Anxiety, panic, or phobia disorder	15%	39%
Intellectual disability	26%	31%
Obsessive‐Compulsive Disorder (OCD)	7%	13%
Depression	1%	8%
Tic/Tourette's disorder	2%	3%
Bipolar disorder	1%	1%
Other medical or psychiatric condition	13%	18%
Gastrointestinal disorder		
Acid reflux/GERD/rumination	8%	6%
Constipation	6%	6%
Encopresis	0%	2%
Food intolerance/sensitivity/celiac disease	2%	1%
Medications		
Stimulant	2%	19%
Anti‐anxiety medication (e.g., benzodiazepine or hypnotics)	1%	14%
Antidepressant	2%	12%
Mood stabilizer	0%	10%
Prescription sleep medication	5%	10%
Antipsychotic/tranquilizer	3%	6%
Hypotensive medication	1%	5%
Anticonvulsant	1%	4%
Other	11%	22%

Abbreviations: GERD, gastroesophageal reflux disease; GI, gastrointestinal.

### Endorsement of gastrointestinal and related symptoms

The prevalence of GI, toileting‐related, mealtime and dietary, and other behaviors, as reported on the ASD‐GIRBI, are indicated in Table S1. Over 80% of participants reported their child experienced one or more GI symptoms in the past 3 months. Among 6–17‐year‐old children, symptoms endorsed by at least a quarter of the sample were flatulence or gas (59%), constipation (54%), abdominal pain (40%), and diarrhea (37%). Among the 3‐5‐year‐old children, these were constipation (64%), flatulence or gas (53%), diarrhea (46%), alternating diarrhea and constipation (33%), and abdominal pain (25%). Strong preferences regarding foods were also very common in the prior 3 months, with the vast majority of children in both age groups (>70%) preferring the same foods at each meal, foods prepared a particular way, avoiding eating a particular type of food group, or strongly preferring certain types of food colors, textures, or temperatures.

Among parents of the 6–17‐year‐old children, 12% reported they were not confident at all in assessing their child's GI pain, 26% reported they were slightly confident, 44% were fairly confident, and only 16% were completely confident. Confidence among the parents of the younger children was even lower (Table 2).

### Item level and exploratory factor analysis

Exploratory factor analysis was carried out only among the 6–17‐year‐old children (*n* = 334) because of insufficient sample size in the younger group (*n* = 110). Two items were dropped for being endorsed by <10% participants (BM frequency > 3 per day and pushing on their own chest/neck/throat). No two items had a pairwise correlation >0.70. Eleven items were removed for lowering the scale's overall Cronbach's alpha (reflux or heartburn, having constipation [type 1 & 2 on Bristol stool chart], diarrhea [types 5, 6, and 7 on Bristol stool chart], <3 BMs per week, inflexibility about mealtimes routines (e.g., times for meals, place settings, seating arrangements, meal locations), refusing foods that requiring lots of chewing, preferring only sweet foods, being on a special diet (e.g., gluten‐free, casein‐free, FODMAPS, GAPS), drinking lots of water with meals, frequent clearing of throat, and avoiding wearing tight clothing or clothing with waistbands). A scree plot using parallel analysis suggested a 7‐factor model fit best for the remaining 43 items (Figure S1), though fit statistics for models ranging from 1 to 10 factors were calculated (Table 3). Fit statistics improved with each factor added to the model, except for the BIC, which increased at the 7‐factor model. The TLI did not reach the acceptable level of 0.9, though this may be a function of average correlations between items not being high (Kenny, 2020). Therefore, the 7‐factor model was selected. An additional five items were dropped from this model for not loading onto any of the factors (abdominal swelling or distension, having ideal stool consistency [types 3 & 4 on Bristol stool scale], rushing to the bathroom for a bowel movement, chewing on non‐food items, difficulty falling or staying asleep), leaving 38 items. The reliability of these 38 items was assessed again, and two more items were dropped for decreasing the scale's Cronbach's alpha (wetting the bed and not remaining seated at the table during mealtimes). A seven‐factor solution also fit these remaining 36 items. Thus, post‐analysis, the tool was reduced from 55 items to these 36, which include the following factors: factor 1‐BM pain; factor 2‐aggressive or disruptive during mealtimes; factor 3‐Particular with foods; factor 4‐abdominal pain and upset stomach; factor 5‐refusing food; factor 6‐constipation and encopresis; factor 7‐motor or other behaviors (Figure S2). The items belonging to each factor are shown in Box 1. Factor loadings for each questionnaire item can be found in Table S2. In total, the seven factors accounted for 43% of the scale's total variance, with each factor accounting for 3%–7%. Eleven of these items were derived from the ATN GI inventory, 11 from the BAMBI, and the remaining 14 from the extant literature or qualitative interviews.

**TABLE 3:** Model fit indices

Model	Number of items	Likelihood Chi square	TLI	RMSR	RMSEA index	RMSEA index 90% CI	BIC
1 factor model	43	3296.01	0.382	0.1	0.092	(0.089, 0.096)	−1701.57
2 factor model	43	2661.84	0.507	0.08	0.082	(0.079, 0.086)	−2091.67
3 factor model	43	2237.68	0.587	0.06	0.075	(0.072, 0.079)	−2277.58
4 factor model	43	1966.79	0.633	0.06	0.071	(0.067, 0.075)	−2316.02
5 factor model	43	1712.54	0.679	0.05	0.066	(0.062, 0.070)	−2343.64
6 factor model	43	1466.72	0.73	0.04	0.060	(0.056, 0.065)	−2368.63
7 factor model	43	1287.06	0.764	0.04	0.056	(0.052, 0.061)	−2333.28
8 factor model	43	1107.24	0.803	0.04	0.051	(0.047, 0.056)	−2303.9
9 factor model	43	981.31	0.827	0.03	0.048	(0.043, 0.053)	−2226.44
10 factor model	43	848.85	0.857	0.03	0.044	(0.038, 0.049)	−2161.32
Final 7 factor model	36	880.73	0.786	0.04	0.060	(0.055, 0.066)	−1437.92

Abbreviations: BIC, Bayesian information criterion; RMSEA, root mean square error of approximation; RMSR, root mean square residual; TLI, Tucker Lewis Index.

**BOX 1:** Factor names and items loading onto each factor of the ASD‐GIRBI^a^

Factor 1: Bowel Movement Pain	Factor 2: Aggressive/Disruptive at Mealtimes	Factor 3: Particular with Foods	Factor 4: Abdominal Pain & Upset Stomach	Factor 5: Refuses food	Factor 6: Constipation & Encopresis	Factor 7: Motor/Other behaviors
Constipation	Cries or screams during mealtimes	Not willing to try new foods	Abdominal pain	Turns their face or body away from food	Constipation	Applying pressure to their abdomen by pushing on it or leaning on furniture
Appear to feel pain when having a BM	Is aggressive during mealtimes (hitting, kicking, scratching others)	Does not accept/ prefer a variety of foods	Nausea, vomiting, or retching/dry heaving	Closes their mouth tightly when food is presented	Alternating constipation and diarrhea	Unusual movements such as thrusting jaw, tilting head, arching back, or twisting neck/body
Become more active after passing a stool	Displays self‐injurious behavior during mealtimes (hitting self, biting self)	Prefers same foods at each meal	Bloating	Spits out food that they have put in their mouth	Incontinence / Lack of voluntary control or urination or defecation	Moaning and groaning
Become less irritable after passing a stool	Is disruptive during mealtimes (pushing/throwing utensils or food)	Prefers food prepared particular way	Flatulence/gas	Stops eating after just a little food	Fecal Retention / complete elimination of stool	Unexplained irritability
	Biting themselves, putting their fist in their mouth, or hurting themselves in other ways	Avoids eating a particular type of food group	Diarrhea	Cries or screams during mealtimes	Stiffen their legs or squeeze their bottom and legs together when they felt need to have a BM	Gritting teeth, wincing, or grimacing for no apparent reason
	Diarrhea	Strongly prefers certain types of food colors, textures, temperatures	Alternating constipation and diarrhea		Stain or soil underwear	
			Pointing to stomach/tummy as if in pain			
			Direct vocalizations of pain (e.g. “tummy hurts” “stomach pain”)			

^a^ Items with a factor loading greater or equal to 0.30 were assigned to a factor. Items could load onto more than one factor.

Abbreviation: ASD‐GIRBI, ASD Gastrointestinal and Related Behaviors Inventory.

### Reliability

Table 4 summarizes the alpha and item‐rest coefficients and item frequency for the final 36 items in the ASD‐GIRBI. The Cronbach's alpha for individual items was 0.87–0.88. The overall alpha for the scale was 0.88, identifying strong internal consistency. Table S3 summarizes correlations between factor scores. Pairwise correlations between factor scores were weak on average, with only two pairs of factor scores reaching a correlation of 0.30 (factors 1 and 4 had a correlation of 0.32; factors 3 and 5 had a correlation of 0.31).

**TABLE 4:** Cronbach's alpha for ASD‐GIRBI scale, among children 6–17 years

Item	Item‐rest corr.	Alpha^d^	Item freq.
Abdominal pain^a^	0.47	0.87	45%
Nausea, vomiting, or retching/dry heaving^a^	0.31	0.88	23%
Bloating^a^	0.46	0.87	27%
Flatulence or gas^c^	0.47	0.87	61%
Diarrhea^c^	0.36	0.88	39%
Constipation^c^	0.43	0.87	58%
Alternating constipation and diarrhea^c^	0.49	0.87	24%
Incontinence/lack of voluntary control or urination or defecation^c^	0.27	0.88	19%
Fecal retention/complete elimination of stool^c^	0.31	0.88	19%
Appear to feel pain when having a BM^a^	0.42	0.87	42%
Stiffen legs/squeeze bottom/legs together when need to have BM^a^	0.50	0.87	39%
Stain or soil underwear^a^	0.40	0.88	48%
Become more active after passing a stool^a^	0.47	0.87	49%
Become less irritable after passing a stool^a^	0.52	0.87	56%
Turns their face or body away from food^b^	0.40	0.88	44%
Closes their mouth tightly when food is presented^b^	0.37	0.88	28%
Spits out food that they have put in their mouth^b^	0.43	0.87	35%
Stops eating after just a little food^c^	0.33	0.88	50%
Cries or screams during mealtimes^b^	0.28	0.88	18%
Is aggressive during mealtimes (e.g., hitting, kicking)^b^	0.36	0.88	14%
Displays self‐injurious behavior during mealtimes^b^	0.33	0.88	10%
Is disruptive during mealtimes (pushing/throwing utensils/food)^b^	0.37	0.88	18%
Is willing to try new foods^b^	0.32	0.88	44%
Accepts or prefers a variety of foods^b^	0.33	0.88	38%
Prefers the same foods at each meal^b^	0.28	0.88	84%
Prefers food prepared in a particular way^b^	0.33	0.88	75%
Prefers to avoid eating a particular type of food group^c^	0.32	0.88	73%
Strongly prefers certain types of food colors/textures/temps^c^	0.39	0.88	74%
Applying pressure to abdomen^a^	0.46	0.87	30%
Unusual movements (e.g., thrusting jaw, tilting head)^a^	0.36	0.88	19%
Moaning or groaning^c^	0.41	0.88	20%
Unexplained irritability^c^	0.44	0.87	43%
Gritting teeth, wincing, or grimacing for no apparent reason^c^	0.38	0.88	23%
Biting themselves, putting fist in mouth, hurting self in other ways^a^	0.29	0.88	16%
Pointing to stomach/tummy as if in pain^c^	0.34	0.88	16%
Direct vocalizations of pain (e.g., “tummy hurts” “stomach pain”)^c^	0.36	0.88	38%

^a^ Derived from ATN GI Inventory.

^b^ Item derived from BAMBI.

^c^ New item derived from literature or interviews.

^d^ Cronbach's alpha for total scale if item is dropped. Cronbach's alpha for total scale if 0.88.

Abbreviations: ASD‐GIRBI, ASD Gastrointestinal and Related Behaviors Inventory; ATN GI, Autism Treatment Network GI; BAMBI, Brief Autism Mealtime Behavior Inventory; BM, bowel movement.

### Convergent validity

Children with a parent‐reported diagnosis of any GI disorder were significantly more likely to have higher clinical scores on factor 1 (BM pain), factor 4 (abdominal pain and upset stomach), factor 6 (constipation and encopresis), and factor 7 (motor or other behaviors) (*p* < 0.05). Children with a parent‐reported diagnosis of acid reflux, GERD, or rumination were significantly more likely to have higher clinical scores on factor 4 (abdominal pain and upset stomach) (*p* < 0.05). Children with a parent‐reported diagnosis of constipation were significantly more likely to have higher clinical scores on factor 1 (BM pain) (Table 5).

**TABLE 5:** Mean differences in ASD‐GIRBI factor scores by parent‐report GI diagnoses

	Any gastrointestinal disorder	Acid reflux/GERD/rumination	Constipation
Factor 1—Bowel movement pain	2.1 versus 1.4*	2.0 versus 1.7	2.8 versus 1.7*
Factor 2—Aggressive/disruptive during mealtimes	1.1 versus 1.0	1.2 versus 1.1	1.1 versus 1.1
Factor 3—Particular with foods	3.8 versus 3.8	4.2 versus 3.8	4.1 versus 3.8
Factor 4—Abdominal pain & upset stomach	3.3 versus 2.1*	3.6 versus 2.5*	2.6 versus 2.6
Factor 5—Refuses food	1.7 versus 1.7	1.8 versus 1.7	1.7 versus 1.7
Factor 6—Constipation & encopresis	2.7 versus 1.5*	2.2 versus 1.9	2.5 versus 1.9
Factor 7—Motor/other behaviors	1.7 versus 1.1*	1.8 versus 1.3	1.7 versus 1.3
Total factor score	14.8 versus 11.3*	15.1 versus 12.6	15.2 versus 12.6*

* Signify mean differences with a *p* < 0.05, according to a *t* test.

Abbreviations: ASD‐GIRBI, ASD Gastrointestinal and Related Behaviors Inventory; GERD, gastroesophageal reflux disease; GI, gastrointestinal.

Correlations between factor scores and CBCL subscale scores were weak to moderate on average (Table S4). Factor 1 (BM pain) was correlated with the CBCL somatic complaints subscale (*r* = 0.45). Factor 2 (aggressive or disruptive during mealtimes) was correlated with the CBCL aggressive behavior subscale (*r* = 0.38). Factor 3 (particular with foods) was correlated with thought problems on the CBCL (*r* = 0.32). Factor 4 (abdominal pain and upset stomach) was correlated with the anxious/depressed (*r* = 0.40) and somatic complaints (*r* = 0.60) subscales on the CBCL. Correlations between factor 5 (refuses foods) and all CBCL subscales were <0.30. Factor 6 (constipation and encopresis) were correlated with somatic complaints (*r* = 0.36) and social problems (*r* = 0.32). Lastly, factor 7 (motor/other behaviors) was correlated with all CBCL subscales (*r* ranging from 0.31 to 0.55).

The mean difference in clinical factor scores was estimated across levels of functional impairment due to GI symptoms (missed school/was late, missed social/family activities, trouble falling/staying asleep). Nearly all factor scores were significantly associated with these three types of functional impairment (*p* < 0.05) (Table S5).

Receiver operating characteristic (ROC) Curves were calculated using cut‐off scores of 1 for each factor score. The factors with the highest AUC for any GI disorder, acid reflux, and constipation were factors 6 (constipation and encopresis), 4 (abdominal pain and upset stomach), and 1 (BM pain), respectively (Table 6). The sensitivity for these items was high (89%–90%) though the specificity ranges from 25% to 34%. Although the mean factor 7 (motor/other behaviors) score was also significantly associated with any GI disorders (Table 5), the AUC and sensitivity for detecting this were slightly better for factor 6 (Table 6).

**TABLE 6:** Sensitivity, specificity, and area under the curve of ASD‐GIRBI clinical factor scores in predicting parent‐report gastrointestinal disorder diagnoses

Parent‐report GI diagnosis	Factor^a^	Sensitivity	Specificity	Area under curve
Any GI disorder	Factor 1 (BM pain)	0.82	0.38	0.64
Any GI disorder	Factor 4 (abdominal pain and upset stomach)	0.86	0.31	0.66
Any GI disorder	Factor 6 (constipation and encopresis)	0.89	0.34	0.70
Any GI disorder	Factor 7 (motor/other behaviors)	0.77	0.46	0.64
Acid reflux/GERD/rumination	Factor 1 (BM pain)	0.90	0.31	0.56
Acid reflux/GERD/rumination	Factor 4 (abdominal pain and upset stomach)	0.90	0.25	0.65
Acid reflux/GERD/rumination	Factor 6 (constipation and encopresis)	0.90	0.26	0.57
Acid reflux/GERD/rumination	Factor 7 (motor/other behaviors)	0.65	0.39	0.58
Constipation	Factor 1 (BM pain)	0.90	0.31	0.71
Constipation	Factor 4 (abdominal pain and upset stomach)	0.75	0.25	0.51
Constipation	Factor 6 (constipation and encopresis)	0.95	0.26	0.61
Constipation	Factor 7 (motor/other behaviors)	0.90	0.41	0.63

^a^ Cut‐off of 1 was used for every factor score to calculate sensitivity, specificity, and AUC.

Abbreviations: ASD‐GIRBI, ASD Gastrointestinal and Related Behaviors Inventory; AUC, area under the curve; BM, bowel movement; GERD, gastroesophageal reflux disease; GI, gastrointestinal.

## DISCUSSION

In this study, we developed a caregiver‐report screener for GI symptoms in children with ASD. This tool includes items assessing GI signs and symptoms, toileting behaviors, mealtime and dietary behaviors, and other non‐GI‐specific behaviors that could indicate GI distress in a non‐verbal or minimally‐verbal child. The resulting 36‐item screener was derived from the ATN GI Inventory, the BAMBI, and novel items created specifically for this measure. We found a 7‐factor solution of the ASD‐GIRBI to be internally consistent for detecting GI symptoms in children with ASD ages 6–17 years of age.

Items indicative of BM pain, abdominal pain and upset stomach, and constipation and encopresis loaded on factors 1, 4, and 6, respectively. Each of these factors was significantly associated with any parent‐reported GI disorder, though the constipation and encopresis factor (factor 6) had the best prediction in terms of AUC. The BM pain factor (factor 1) was also significantly associated with constipation and has the highest AUC. The abdominal pain and upset stomach factor (factor 6) was the only factor across all seven that was significantly associated with parent‐report of acid reflux or rumination and had the highest AUC as well.

Lastly, factor 7 consisted of motor or other non‐specific behaviors that may not always be intuitively linked to the presence of GI symptoms. These behaviors, such as irritability, moaning and groaning, or unusual movements, could indicate several things in a child with ASD. These behaviors were significantly associated with constipation, although the sensitivity and area under the curve for detecting this diagnosis were not as high as those of factor 6. These behavioral items may play a role in helping identify GI disorders in children with ASD. Over a third reported they were either slightly or not at all confident in assessing their child's GI pain.

Our ROC analyses suggest that the BM pain may be especially useful for detecting constipation, the abdominal pain and upset stomach factor may be best for predicting acid reflux, and the constipation and encopresis factor may be good at identifying children with possible constipation as well as any GI disorder. These three factors (factors 1, 4, and 6) consist of just 18 items in total. The addition of factor 7 (motor/other behaviors) items brings this total to 23 items. We were particularly interested in developing a sensitive screener since we are most concerned with identifying children with GI symptoms who might otherwise go undetected. Therefore, the low specificities associated with these factors are acceptable but speak to the need for further refinement and validation of this tool, especially given the potential for false positives, which could lead to unnecessary further evaluation and distress about the possibility of a GI disorder.

The three food‐ or mealtime‐related factors (aggressive or disruptive during mealtimes, particular with foods, refuses foods) were not significantly associated with parent‐report of GI diagnoses in the child. This may speak to the multifactorial nature of feeding behaviors among children with ASD. Feeding problems in ASD have been proposed to be related to sensory sensitivities, restricted or obsessive interests, fear of novelty, potential food intolerances, among other reasons (Cumine et al., 2009; Ledford & Gast, 2006; Maenner et al., 2020). Limited diets may impact nutritional needs, such as adequate fiber consumption, which may result in GI symptoms such as constipation (Ibrahim et al., 2009; Kuddo & Nelson, 2003; Levin & Carr, 2001). However, given the multifactorial nature of these dietary behaviors, they may not be as useful for detecting GI symptoms. More research is needed to clarify this, however. We also note that the diarrhea item loaded onto factor 2 (aggressive or disruptive during mealtimes). Though the loading was not very strong (0.32), it does suggest that diarrhea or urgency to use the toilet during mealtimes might lead to disruptive behaviors. However, this is not possible to confirm in the present study.

While not part of the psychometric analysis presented here, the ASD‐GIRBI also includes items on the duration of symptoms, medications the child is taking, and how the child's GI symptoms affect their functioning. These questions and those on diet and mealtimes may serve as useful contextual information for parents to share with researchers and clinicians. We found that each factor on the ASD‐GIRBI was significantly associated with greater levels of functional impairment at school, in social settings, or with sleeping, or all of the above in many cases. This highlights how GI and related symptoms may impact a child's functioning across multiple settings and influence other health comorbidities. It also supports the value of including all factors in the ASD‐GIRBI.

Although correlations between factor scores and the CBCL subscales were weak to moderate, they were all positive in magnitude, meaning having worse GI or related symptoms is associated with specific problem behaviors and mental health symptoms on the CBCL. As expected, the CBCL somatic complaints subscale was associated with the greatest number of factors on the ASD‐GIRBI. Given that none of the CBCL subscales measure GI symptoms, we would not expect to find very strong associations. Seeing moderate, positive correlations with somatic complaints, aggressive behaviors, and anxiety/depression, for example, provides some evidence of convergent validity and supports the need to include all factors in the ASD‐GIRBI.

There were some limitations to this study. Perhaps most importantly, we did not have a gold‐standard measure of GI symptoms against which to compare our tool. We relied on parent‐report of GI diagnoses to calculate sensitivity and specificity. Ideally, every child in our study would have been assessed for a GI disorder by a physician; however, this was not feasible in this current study. Even if feasible, the more critical issue is that there is currently no gold‐standard approach to assessing GI disorders in people with ASD. Even a trained gastroenterologist may misclassify someone as not having a GI disorder if the patient or a proxy respondent cannot accurately report symptoms. Indeed, rates of parent‐reported GI diagnoses in this study may be underestimates of the true prevalence of disorders due to under‐evaluation and under‐diagnosis. However, this remains an important direction for future work. Comparing the validity and reliability of the ASD‐GIRBI with other frequently used tools in ASD studies, such as the Rome Criteria (Ferguson et al., 2017), is also worthwhile.

Another limitation of this study is that due to insufficient sample size in children under 6 years old, we were only able to carry out the psychometric analysis among children 6–17 years old. The performance of individual items and the factor model would likely differ in this younger group, considering how age modifies the association between GI symptoms and externalizing and internalizing behaviors among children with ASD (Ferguson et al., 2019). Next, our response rate was relatively low, at 23%, which is not uncommon in research, particularly internet‐based research. Given this response rate paired with recruitment from a single research registry, our sample may not adequately represent all children with ASD, potentially limiting our findings' generalizability. Similarly, the frequency of GI and related behaviors from this study should not be considered representative of the underlying ASD population, as parents with children who have more frequent or severe GI symptoms are more likely to participate in studies like this. Indeed, the study title “Autism GI symptoms questionnaire” was included in recruitment material, which likely motivated potential participants with a chronic or more severe history of GI symptoms to participate. We, unfortunately, did not have demographic or clinical information on families who did not wish to enroll in the study, so we cannot assess how similar or different the non‐participants are from our study sample. A previous manuscript has examined predictors of consent to this ASD registry and found some key differences. Black/African‐American families and those who lived geographically further away from the clinic were less likely to participate in the registry (Kalb et al., 2019). Therefore, it seems likely that participants who joined this study were different from those in the underlying clinic population. Further, the families that participated in the qualitative interviews may also not fully represent the underlying population of families with a child with autism and co‐occurring GI symptoms.

Our study also had several strengths. First, the ASD‐GIRBI is based on two existing standardized measures and includes new items based on qualitative interviews with parents of children with ASD and the extant literature. Therefore, we feel confident that this stakeholder‐informed tool has a high degree of content validity, especially since qualitative parental reports were included in the item development process. The parent‐report nature of the ASD‐GIRBI is useful for epidemiologic studies because it is inexpensive and easy to administer to study participants. This study is only the second to carry out a psychometric assessment of a GI assessment tool for individuals with ASD, and several analyses supported the concurrent validity of our novel measure. The first psychometric study was published in 2019, was also based on the ATN‐GI Inventory, and has items in common with the tool analyzed in the present study (Margolis et al., 2019).

While the need for more accurate assessment of GI issues in ASD is receiving increasing attention, there is still much work to do. Parent‐ and self‐report tools need further validation efforts in diverse groups of people with ASD. Tools developed for research purposes, such as this one, may also be useful in clinical settings. In an equitable world, multiple versions of a GI measure such as the ASD‐GIRBI would exist to allow the highest possible accuracy and reliability of GI symptom estimates within a population with heterogeneous neurodevelopmental functioning. Ideally, a patient or participant would complete a short standardized neurodevelopmental assessment to determine which GI measure they should receive. Upon completion of that specific GI measure (by patient/participant, parent/caregiver, or another proxy) and depending on the results of that measure, the individual might be triaged for further evaluation and/or receive treatment for their GI distress. The additional evaluation might include other types of data (e.g., heart rate variability, stool sample, clinician examination) to capture GI symptoms more accurately in this population. Individuals with ASD deserve to have their GI symptoms recognized and treated with the same quality as typically developing individuals.

## ETHICS STATEMENT

The Johns Hopkins University Bloomberg School approved the study of Public Health institutional review board.

## Supporting information


**Appendix** **S1:** Supporting Information

**Appendix** **S2:** Supporting Information

## Data Availability

The data that support the findings of this study are available from the corresponding author upon reasonable request.
