# Supplementary material for: Characteristics of the autism spectrum disorder gastrointestinal and related behaviors inventory in children
Source: Autism Res. 2022 Mar 18;15(6):1142–55. doi: 10.1002/aur.2707 (PMC9262638; doi:10.1002/aur.2707)
Supplement: Supplementary file 1 — Appendix S1: Supporting Information [file AUR-15-1142-s001.docx]

Autism Spectrum Disorder Gastrointestinal and Related Behaviors Inventory

**Instructions for Parents/Primary Caregivers:**
The purpose of this questionnaire is to assess the presence of gastrointestinal (GI) symptoms and related issues in children with autism spectrum disorder. This questionnaire may take up to 30 minutes to complete. The questionnaire should only be completed by the child’s parent or another primary caregiver. There are no right or wrong answers to these questions, but please answer each question to the best of your ability. You may choose not to answer questions that make you feel uncomfortable. 
Please note that all information will be kept strictly confidential.

Date questionnaire completed (DD/MM/YYYY)

_________________________________

Please indicate your relationship to the child:

- Mother
- Father
- Other primary caregiver

If Other, please describe:

_________________________________

Please indicate your highest level of education completed.

- Some high school
- High school graduate or GED
- Some college or associate degree education
- College or associate degree
- Graduate education

Please indicate your child's biological sex at birth.

- Female
- Male

Please indicate your child's gender identity.

- Female
- Male
- Transgender
- Non-binary, Gender-queer, or Gender-fluid
- Other

Please indicate your child's race/ethnicity (select all that apply).

- American Indian or Alaska Native
- Asian
- Black or African American
- Hispanic or Latino
- Native Hawaiian or Other Pacific Islander
- White

Please indicate your child's current age in years.

_________________________________

Has your child been diagnosed with any of the following conditions?

|  | No | Yes | Not Sure |
| --- | --- | --- | --- |
| Any gastrointestinal disorder |  |  |  |
| Epilepsy/Seizure disorder |  |  |  |
| Intellectual disability |  |  |  |
| ADD/ADHD |  |  |  |
| Sensory processing disorder |  |  |  |
| Anxiety, panic, or phobia disorder |  |  |  |
| OCD |  |  |  |
| Tic/Tourette's disorder |  |  |  |
| Depression |  |  |  |
| Bipolar Disorder |  |  |  |
| Sleep disorder |  |  |  |
| Autoimmune disorder |  |  |  |
| Allergies or Asthma |  |  |  |
| Other |  |  |  |

If your child has been diagnosed with a gastrointestinal disorder, which gastrointestinal disorder(s) has your child been diagnosed with?

_________________________________

_________________________________

_________________________________

_________________________________

If other, please specify which other psychiatric/behavioral/medical condition(s) has your child been diagnosed with?

_________________________________

_________________________________

_________________________________

_________________________________

In the last 3 months, has your child experienced any of the following gastrointestinal symptoms?

|  | No | Yes | Not Sure |
| --- | --- | --- | --- |
| Abdominal Pain |  |  |  |
| Nausea, Vomiting, or Retching/Dry Heaving |  |  |  |
| Reflux or Heartburn |  |  |  |
| Abdominal swelling or distension |  |  |  |
| Bloating |  |  |  |
| Flatulence or Gas |  |  |  |
| Diarrhea |  |  |  |
| Constipation |  |  |  |
| Alternating constipation and diarrhea |  |  |  |
| Incontinence / Lack of voluntary control over urination or defecation |  |  |  |
| Fecal retention / incomplete elimination of stool |  |  |  |

How long has your child experienced these symptoms?

|  | Within the last 3 months only | 3-5 months | 6-11 months | 1 year or longer | Not Sure | NA, has not experienced symptom in last 3 months |
| --- | --- | --- | --- | --- | --- | --- |
| Abdominal pain |  |  |  |  |  |  |
| Nausea, Vomiting, Retching/Dry Heaving |  |  |  |  |  |  |
| Reflux or Heartburn |  |  |  |  |  |  |
| Abdominal swelling or distension |  |  |  |  |  |  |
| Bloating |  |  |  |  |  |  |
| Flatulence or Gas |  |  |  |  |  |  |
| Diarrhea |  |  |  |  |  |  |
| Constipation |  |  |  |  |  |  |
| Alternating constipation and diarrhea |  |  |  |  |  |  |
| Incontinence / Lack of voluntary control over urination or defecation |  |  |  |  |  |  |
| Fecal retention / incomplete elimination of stool |  |  |  |  |  |  |

In the last 3 months of your child having these symptoms...

|  | No | Yes | Not Sure | NA, has not experienced any symptoms in last 3 months |
| --- | --- | --- | --- | --- |
| Do the symptoms get better after having a bowel movement (pooping)? |  |  |  |  |
| Do the symptoms occur before eating or when hungry? |  |  |  |  |
| Do the symptoms improve after your child eats? |  |  |  |  |
| Do the symptoms worsen after your child eats? |  |  |  |  |
| Has your child had trouble gaining weight? |  |  |  |  |

It can be difficult for parents/caregivers to accurately assess their child's pain level. How confident do you feel in your ability to assess your child's gastrointestinal pain?

- Not confident at all
- Slightly confident
- Fairly confident
- Completely confident

In the last 3 months, how often did your child usually have a bowel movement (BM), i.e. pooping?

- Once a day
- Less than once a day
- More than once a day
- Not sure

If you selected less than once a day, in the last 3 months, how often did your child usually have a bowel movement (BM), i.e. pooping?

- 3-6 times per week
- 1-2 times per week
- Less than once per week
- Not sure

If you selected more than once a day, in the last 3 months, how often did your child usually have a bowel movement (BM), i.e. pooping?

- 2-3 times per day
- 3+ times per day
- Not sure


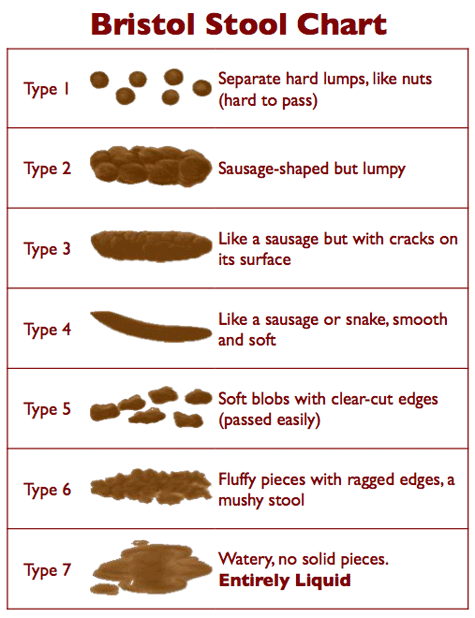


In the last 3 months, what was your child's stool usually like? Of the 7 options below, please order them from most commonly like your child's stool to least commonly like your child's stool. 
Write a 1 next to the most common stool, a 2 next to the second most common down to stool, etc. all the way to 7, the least common type of stool.

Please see the image above for reference.

______ Type 1: Separate hard lumps

______ Type 2: Lumpy and sausage like

______ Type 3: A sausage shape with cracks in the surface

______ Type 4: Like a smooth, soft sausage or snake

______ Type 5: Soft blobs with clear-cut edges

______ Type 6: Mushy consistency with ragged edges

______ Type 7: Liquid consistency with no solid pieces

In the last 3 months, did your child...

|  | No | Yes | Not Sure |
| --- | --- | --- | --- |
| ...Appear to feel pain when having a BM? |  |  |  |
| ...Rush to the bathroom for a BM? |  |  |  |
| ...Stiffen their legs or squeeze their bottom and legs together when they felt the need to have a BM? |  |  |  |
| ...Stain or soil underwear? |  |  |  |
| ...Wet the bed? |  |  |  |
| ...Become more active after passing a stool? |  |  |  |
| ...Become less irritable after passing a stool? |  |  |  |

Think about mealtimes with your child over the past 3 months. Rate the following items according to how often each occurs, using the following scale. My child...

|  | Never/Rarely | Several times per month | 1-2 times per week | 3 or more times per week | Not Sure |
| --- | --- | --- | --- | --- | --- |
| ...Turns their face or body away from food |  |  |  |  |  |
| ...Closes their mouth tightly when food is presented |  |  |  |  |  |
| ...Spits out food that they have put in their mouth |  |  |  |  |  |
| ...Stops eating after just a little food |  |  |  |  |  |
| ...Remains seated at the table until the meal is finished |  |  |  |  |  |
| ...Cries or screams during mealtimes |  |  |  |  |  |
| ...Is aggressive during mealtimes (hitting, kicking, scratching others) |  |  |  |  |  |
| ...Displays self-injurious behavior during mealtimes (hitting self, biting self) |  |  |  |  |  |
| ...Is disruptive during mealtimes (pushing/throwing utensils or food) |  |  |  |  |  |
| ...Is flexible about mealtime routines (e.g. times for meals, place settings, seating arrangements, meal locations) |  |  |  |  |  |
| ...Is willing to try new foods |  |  |  |  |  |
| ...Accepts or prefers a variety of foods |  |  |  |  |  |

Think about mealtimes with your child over the past 3 months. Rate the following items according to how often each occurs, using the following scale. My child...

|  | Never/Rarely | Several times per month | 1-2 times per week | 3 or more times per week | Not Sure |
| --- | --- | --- | --- | --- | --- |
| ...Prefers the same foods at each meal |  |  |  |  |  |
| ...Prefers food prepared in a particular way (e.g. eats mostly fried foods, cold cereals, raw vegetables) |  |  |  |  |  |
| ...Prefers to avoid eating a particular type of food group (e.g. vegetables, meats, dairy) |  |  |  |  |  |
| ...Strongly prefers certain types of food colors, textures, or temperatures |  |  |  |  |  |
| ...Refuses to eat foods that require a lot of chewing (e.g. eats only soft or pureed foods) |  |  |  |  |  |
| ...Prefers only sweet foods (e.g. candy, sugary cereals) |  |  |  |  |  |
| ...Is on a special diet (e.g. gluten free, casein free, FODMAPS, GAPS) |  |  |  |  |  |
| ...Drink lots of water with meals |  |  |  |  |  |

In the last 3 months, please indicate whether or not you've observed the following behaviors in your child?

|  | No | Yes | Not Sure |
| --- | --- | --- | --- |
| Pushing on their own chest/neck/throat |  |  |  |
| Applying pressure to their abdomen by pushing on it or leaning on furniture |  |  |  |
| Unusual movements such as thrusting jaw, tilting head, arching back, or twisting neck/body |  |  |  |
| Frequent clearing of throat, swallowing, coughing, gagging, choking, or throat sounds wet or gurgly |  |  |  |
| Moaning or groaning for no apparent reason |  |  |  |
| Unexplained irritability, agitation, aggression, or screaming |  |  |  |
| Gritting teeth, wincing, or grimacing for no obvious reason |  |  |  |
| Biting themselves, putting their fist in their mouth, or hurting themselves in other ways |  |  |  |
| Avoid wearing tight clothing or clothing with waistbands |  |  |  |
| Chewing on shirts, eating non-edible objects |  |  |  |
| Pointing to stomach/tummy as if in pain |  |  |  |
| Direct verbalizations of pain (e.g. "tummy hurts" "stomach pain") |  |  |  |
| Difficulty falling asleep or staying asleep |  |  |  |

Please select whether or not your child currently takes any of the following medications.

|  | No | Yes | Not Sure |
| --- | --- | --- | --- |
| Antidepressant |  |  |  |
| Antipsychotic or Tranquilizer |  |  |  |
| Anti-anxiety medication (e.g. benzodiazepine or hypnotics) |  |  |  |
| Mood stabilizer |  |  |  |
| Stimulant |  |  |  |
| Anticonvulsant |  |  |  |
| Prescription sleeping medication |  |  |  |
| Hypotensive medication |  |  |  |
| Other |  |  |  |

If other, please specify:

_________________________________

In the last 3 months, have your child's GI symptoms led them to...

|  | Rarely/Never | Several times per month | 1-2 times per week | 3 or more times per week | Not Sure |
| --- | --- | --- | --- | --- | --- |
| Get to school late, miss school, or leave school early (including missing parts of class)? |  |  |  |  |  |
| Miss social or family activities? |  |  |  |  |  |
| Have trouble falling asleep or staying asleep? |  |  |  |  |  |

This GI questionnaire was designed using items from the Autism Treatment Network GI Inventory and the Brief Autism Mealtime Behaviors Inventory (BAMBI). This Network activity was supported by Autism Speaks and cooperative agreement UA3 MC11054 through the U.S. Department of Health and Human Services, Health Resources and Services Administration, Maternal and Child Health Research Program to the Massachusetts General Hospital. This work was conducted through the Autism Speaks Autism Treatment Network. This work was funded by the Wendy Klag Center for Autism and Developmental Disabilities.
