# Supplementary material for: Characteristics of the autism spectrum disorder gastrointestinal and related behaviors inventory in children
Source: Autism Res. 2022 Mar 18;15(6):1142–55. doi: 10.1002/aur.2707 (PMC9262638; doi:10.1002/aur.2707)

**Table S1. Gastrointestinal Symptoms, Toileting-Related Behaviors, Mealtime/Dietary Behaviors, and Other Behaviors in Last three Months, Stratified by Age (%)**

|  | Ages 3-5 years  (n=110) | Ages 6-17 years  (n=334) |
| --- | --- | --- |
| Gastrointestinal Symptoms |  |  |
| Abdominal pain | 25% | 40% |
| Nausea, vomiting, or retching/dry heaving | 24% | 22% |
| Reflux or heartburn | 15% | 14% |
| Abdominal swelling or distention | 12% | 13% |
| Bloating | 22% | 24% |
| Flatulence or gas | 53% | 59% |
| Diarrhea | 46% | 37% |
| Constipation | 64% | 54% |
| Alternating diarrhea and constipation | 33% | 22% |
| Incontinence / Lack of voluntary control or urination or defecation | 24% | 18% |
| Fecal Retention / complete elimination of stool | 22% | 16% |
| Any of the above | 83% | 83% |
| Bristol Stool Chart – Constipation (Types 1 & 2) | 53% | 51% |
| Bristol Stool Chart – Diarrhea (Types 5, 6, 7) | 34% | 25% |
| Bristol Stool Chart – Ideal (Types 3 & 4) | 49% | 57% |
| Average BM more than 3 per day | 5% | 3% |
| Average BM less than three per week | 15% | 17% |
| Toileting-Related Behaviors |  |  |
| Appear to feel pain when having a BM | 40% | 36% |
| Rush to the bathroom for a BM | 35% | 52% |
| Stiffen their legs or squeeze their bottom and legs together when they  felt need to have a BM | 45% | 32% |
| Stain or soil underwear | 58% | 47% |
| Wet the bed | 43% | 29% |
| Become more active after passing a stool | 53% | 37% |
| Become less irritable after passing a stool | 48% | 45% |
| Mealtime and Dietary Behaviors - several times per month or more |  |  |
| Turns their face or body away from food | 68% | 43% |
| Closes their mouth tightly when food is presented | 57% | 27% |
| Spits out food that they have put in their mouth | 56% | 34% |
| Stops eating after just a little food | 66% | 49% |
| Remains seated at the table until the meal at finished | 45% | 62% |
| Cries or screams during mealtimes | 41% | 18% |
| Is aggressive during mealtimes (hitting, kicking, scratching others) | 25% | 14% |
| Displays self-injurious behavior during mealtimes (hitting self, biting  self) | 12% | 10% |
| Is disruptive during mealtimes (pushing/throwing utensils or food) | 41% | 18% |
| Is flexible about mealtime routines (e.g. times for meals, place settings,  seating arrangements, meal locations) | 74% | 76% |
| Is willing to try new foods | 35% | 55% |
| Accepts or prefers a variety of foods | 45% | 62% |
| Prefers the same foods at each meal | 89% | 83% |
| Prefers food prepared in a particular way | 78% | 72% |
| Prefers to avoid eating a particular types of food group (e.g. vegetables,  meats, dairy) | 76% | 71% |
| Strongly prefers certain types of food colors, textures, or temperatures | 70% | 73% |
| Refuses to eat foods that require a lot of chewing (e.g. eats only soft or  pureed foods) | 29% | 19% |
| Prefers only sweet foods (e.g. candy, sugary cereals) | 54% | 47% |
| Is on a special diet (e.g. gluten free, casein free, FODMAPS, GAPS) | 14% | 15% |
| Drinks lots of water with meals | 71% | 69% |
| Other Behaviors |  |  |
| Pushing on their own chest/neck/throat | 6% | 8% |
| Applying pressure to their abdomen by pushing on it or leaning on  furniture | 37% | 28% |
| Unusual movements such as thrusting jaw, tilting head, arching back, or  twisting neck/body | 17% | 18% |
| Frequent clearing of throat, swallowing, coughing, gagging, choking, or  throat sounds wet or gurgly | 25% | 34% |
| Moaning or groaning for no apparent reason | 18% | 19% |
| Unexplained irritability, agitation, aggression, or screaming | 53% | 41% |
| Gritting teeth, wincing, or grimacing for no apparent reason | 24% | 22% |
| Biting themselves, putting their fist in their mouth, or hurting  themselves in other ways | 10% | 16% |
| Avoid wearing tight clothing or clothing with waistbands | 22% | 25% |
| Chewing on shirts, eating non-edible objects | 43% | 39% |
| Pointing to stomach/tummy as if in pain | 15% | 15% |
| Direct vocalizations of pain (e.g. “tummy hurts” “stomach pain”) | 26% | 37% |
| Difficulty falling asleep or staying asleep | 47% | 53% |

**Table S2. Factor Loadings of ASD-GIRB Items in Seven-Factor Exploratory Factor Model**

|  | Factor 1 | | Factor 2 | | Factor 3 | | Factor 4 | | Factor 5 | | Factor 6 | | Factor 7 |
| --- | --- | --- | --- | --- | --- | --- | --- | --- | --- | --- | --- | --- | --- |
|  | Bowel Movement Pain | Aggressive/disruptive during mealtime | | Particular with foods | | Abdominal pain and upset stomach | | Refuses food | | Constipation & Encopresis | | Motor/  Other Behavior | |
| Abdominal pain | 0.05 | -0.02 | | -0.03 | | **0.80** | | 0.02 | | 0.01 | | -0.01 | |
| Nausea, vomiting, or retching/dry heaving | -0.18 | 0.07 | | 0.02 | | **0.50** | | 0.09 | | 0.05 | | 0.01 | |
| Bloating | 0.25 | -0.07 | | 0.10 | | **0.39** | | -0.12 | | 0.13 | | 0.18 | |
| Flatulence or gas | 0.25 | 0.02 | | 0.06 | | **0.43** | | -0.02 | | 0.12 | | -0.05 | |
| Diarrhea | -0.14 | **0.32** | | 0.09 | | **0.36** | | 0.01 | | 0.21 | | -0.22 | |
| Constipation | **0.49** | -0.05 | | 0.03 | | 0.17 | | -0.14 | | **0.33** | | -0.11 | |
| Alternating constipation and diarrhea | 0.04 | 0.28 | | 0.17 | | **0.31** | | -0.12 | | **0.36** | | -0.19 | |
| Incontinence / Lack of voluntary control or urination or defecation | -0.15 | 0.11 | | -0.10 | | -0.05 | | 0.03 | | **0.62** | | 0.13 | |
| Fecal Retention / complete elimination of stool | 0.23 | -0.08 | | 0.01 | | -0.02 | | -0.01 | | **0.54** | | -0.14 | |
| Appear to feel pain when having a BM | **0.42** | -0.08 | | -0.02 | | 0.27 | | 0.02 | | 0.12 | | 0.01 | |
| Stiffen their legs or squeeze their bottom and legs together when they felt need to have a BM | 0.23 | -0.11 | | 0.04 | | -0.05 | | 0.20 | | **0.52** | | 0.17 | |
| Stain or soil underwear | -0.08 | 0.05 | | 0.03 | | 0.03 | | 0.09 | | **0.57** | | 0.12 | |
| Become more active after passing a stool | **0.66** | 0.02 | | -0.01 | | 0.01 | | 0.11 | | 0.07 | | 0.06 | |
| Become less irritable after passing a stool | **0.86** | 0.11 | | 0.04 | | 0.02 | | 0.04 | | -0.08 | | 0.03 | |
| Turns their face or body away from food | 0.07 | -0.01 | | 0.04 | | 0.04 | | **0.81** | | -0.01 | | -0.07 | |
| Closes their mouth tightly when food is presented | 0.05 | 0.17 | | 0.14 | | -0.13 | | **0.56** | | 0.04 | | -0.01 | |
| Spits out food that they have put in their mouth | -0.06 | 0.02 | | 0.06 | | 0.10 | | **0.56** | | 0.08 | | 0.17 | |
| Stops eating after just a little food | 0.01 | -0.01 | | -0.01 | | 0.06 | | **0.54** | | 0.09 | | 0.00 | |
| Cries or screams during mealtimes | -0.07 | **0.36** | | -0.05 | | 0.07 | | **0.35** | | -0.06 | | 0.04 | |
| Is aggressive during mealtimes (hitting, kicking, scratching others) | 0.07 | **0.77** | | -0.05 | | 0.01 | | 0.05 | | -0.07 | | 0.03 | |
| Displays self-injurious behavior during mealtimes (hitting self, biting self) | 0.03 | **0.64** | | 0.07 | | -0.10 | | -0.07 | | 0.06 | | 0.10 | |
| Is disruptive during mealtimes (pushing/throwing utensils or food) | 0.08 | **0.64** | | -0.03 | | -0.03 | | 0.16 | | 0.04 | | -0.07 | |
| Is willing to try new foods | -0.16 | 0.06 | | **0.52** | | 0.09 | | 0.13 | | -0.06 | | 0.06 | |
| Accepts or prefers a variety of foods | -0.15 | -0.07 | | **0.53** | | 0.17 | | 0.11 | | 0.01 | | 0.00 | |
| Prefers the same foods at each meal | -0.01 | 0.06 | | **0.57** | | -0.01 | | 0.00 | | 0.01 | | -0.11 | |
| Prefers food prepared in a particular way (e.g. eats mostly fried foods, cold cereals, raw vegetables) | 0.05 | 0.02 | | **0.55** | | -0.01 | | 0.02 | | 0.07 | | -0.11 | |
| Prefers to avoid eating a particular types of food group (e.g. vegetables, meats, dairy) | 0.04 | -0.08 | | **0.76** | | -0.12 | | 0.03 | | -0.01 | | 0.02 | |
| Strongly prefers certain types of food colors, textures, or temperatures | 0.08 | 0.04 | | **0.64** | | 0.02 | | -0.03 | | -0.09 | | 0.11 | |
| Applying pressure to their abdomen by pushing on it or leaning on furniture | 0.14 | 0.02 | | 0.06 | | 0.29 | | -0.03 | | 0.10 | | **0.38** | |
| Unusual movements such as thrusting jaw, tilting head, arching back, or twisting neck/body | 0.06 | 0.20 | | 0.12 | | 0.05 | | -0.12 | | 0.13 | | **0.37** | |
| Moaning or groaning | 0.00 | 0.17 | | 0.03 | | 0.28 | | 0.07 | | 0.00 | | **0.31** | |
| Unexplained irritability | 0.23 | 0.27 | | 0.01 | | 0.09 | | 0.02 | | 0.00 | | **0.34** | |
| Gritting teeth, wincing, or grimacing for no apparent reason | 0.04 | 0.08 | | 0.02 | | 0.06 | | 0.03 | | 0.16 | | **0.54** | |
| Biting themselves, putting their fist in their mouth, or hurting themselves in other ways | -0.02 | **0.42** | | 0.04 | | -0.07 | | -0.05 | | 0.11 | | 0.25 | |
| Pointing to stomach/tummy as if in pain | 0.02 | -0.09 | | 0.06 | | **0.43** | | 0.03 | | 0.01 | | 0.18 | |
| Direct vocalizations of pain (e.g. “tummy hurts” “stomach pain”) | 0.06 | -0.07 | | -0.01 | | **0.70** | | 0.05 | | -0.15 | | 0.07 | |

*Note: Factor loadings greater or equal to 0.30 are* ***bolded****.*

**Table S3. Correlations between ASD-GIRB Factors, among Children 6-17 Years**

|  | Factor 1 | Factor 2 | Factor 3 | Factor 4 | Factor 5 | Factor 6 | Factor 7 |
| --- | --- | --- | --- | --- | --- | --- | --- |
| Factor 1 | 1.00 | 0.13 | 0.19 | **0.32** | 0.11 | 0.28 | 0.17 |
| Factor 2 | 0.13 | 1.00 | 0.13 | 0.10 | 0.27 | 0.22 | 0.18 |
| Factor 3 | 0.19 | 0.13 | 1.00 | 0.20 | **0.31** | 0.15 | 0.09 |
| Factor 4 | **0.32** | 0.10 | 0.20 | 1.00 | 0.10 | 0.28 | 0.11 |
| Factor 5 | 0.11 | 0.27 | **0.31** | 0.10 | 1.00 | 0.08 | 0.15 |
| Factor 6 | 0.28 | 0.22 | 0.15 | 0.28 | 0.08 | 1.00 | 0.10 |
| Factor 7 | 0.17 | 0.18 | 0.09 | 0.11 | 0.15 | 0.10 | 1.00 |

Factor 1 - Bowel Movement Pain; Factor 2 – Aggressive/Disruptive during Mealtimes; Factor 3 - Particular with foods; Factor 4 - Abdominal Pain & Upset Stomach; Factor 5 – Refuses Food; Factor 6 – Constipation & Encopresis; Factor 7 – Motor/Other behaviors

*Note: Moderate-sized correlations (r>=0.30) are bolded*

**Table S4. Correlation between ASD-GIRB Factor Scores and CBCL Subscales, among Children 6-17 Years**

|  |  |  |  |  |  |  |  |  |
| --- | --- | --- | --- | --- | --- | --- | --- | --- |
|  | Anxious/  Depressed | Withdrawn Depression | Rule Breaking Behavior | Somatic Complaints | Aggressive Behavior | Social Problems | Thought Problems | Attention Problems |
| Factor 1 | 0.15 | 0.15 | 0.06 | **0.45** | 0.18 | 0.22 | 0.20 | 0.10 |
| Factor 2 | 0.11 | 0.17 | 0.29 | 0.20 | **0.38** | 0.14 | 0.25 | 0.21 |
| Factor 3 | 0.20 | 0.19 | 0.10 | 0.15 | 0.20 | 0.11 | **0.32** | 0.16 |
| Factor 4 | **0.40** | 0.24 | 0.23 | **0.60** | 0.26 | 0.29 | 0.26 | 0.21 |
| Factor 5 | 0.18 | 0.07 | 0.15 | 0.10 | 0.27 | 0.07 | 0.05 | 0.16 |
| Factor 6 | 0.16 | 0.23 | 0.16 | **0.36** | 0.19 | **0.32** | 0.21 | 0.26 |
| Factor 7 | **0.43** | **0.36** | **0.31** | **0.43** | **0.55** | **0.34** | **0.50** | **0.42** |

Factor 1 - Bowel Movement Pain; Factor 2 – Aggressive/Disruptive during Mealtimes; Factor 3 - Particular with foods; Factor 4 - Abdominal Pain & Upset Stomach; Factor 5 – Refuses Food; Factor 6 – Constipation & Encopresis; Factor 7 – Motor/Other behaviors

*Note:* *Moderate-sized correlations (r>=0.30) are* ***bolded****.*

**Table S5. Mean Difference in ASD-GIRB Factor Scores by Functional Impairment**

|  |  | | |
| --- | --- | --- | --- |
| GI Tool Factor Scale Scores | Missed School/Was Late (n=50) | Missed Social/Family Activities (n=56) | Trouble Falling/Staying Asleep (n=130) |
| Factor 1 - Bowel Movement Pain | 2.6 vs. 1.6* | 2.4 vs. 1.6* | 2.1 vs. 1.4* |
| Factor 2 - Aggressive/disruptive during mealtimes | 1.5 vs. 1.1 | 1.5 vs. 1.0 | 1.5 vs. 0.8* |
| Factor 3 - Particular with foods | 4.1 vs. 3.7 | 4.3 vs. 3.7* | 3.9 vs. 3.6 |
| Factor 4 - Abdominal Pain & Upset Stomach | 4.2 vs. 2.3* | 3.6 vs. 2.3* | 3.4 vs. 1.9* |
| Factor 5 - Refuses Food | 2.4 vs. 1.6* | 2.2 vs. 1.6* | 2.0 vs. 1.5* |
| Factor 6 - Constipation & Encopresis | 3.0 vs. 1.7* | 2.9 vs. 1.7* | 2.4 vs. 1.5* |
| Factor 7 - Motor/Other behaviors | 2.2 vs. 1.1* | 1.6 vs. 1.2* | 1.6 vs. 0.9* |
| Total Factor Score | 18.0 vs 11.9* | 16.5 vs 11.9* | 15.2 vs 10.7* |

Table S5 shows the mean factor scale in children by presence versus absence of functional impairment. * denotes p<0.05 for t-test of mean difference.

**Figure S1. Scree Plot Indicated Possible Seven-Factor Solution**


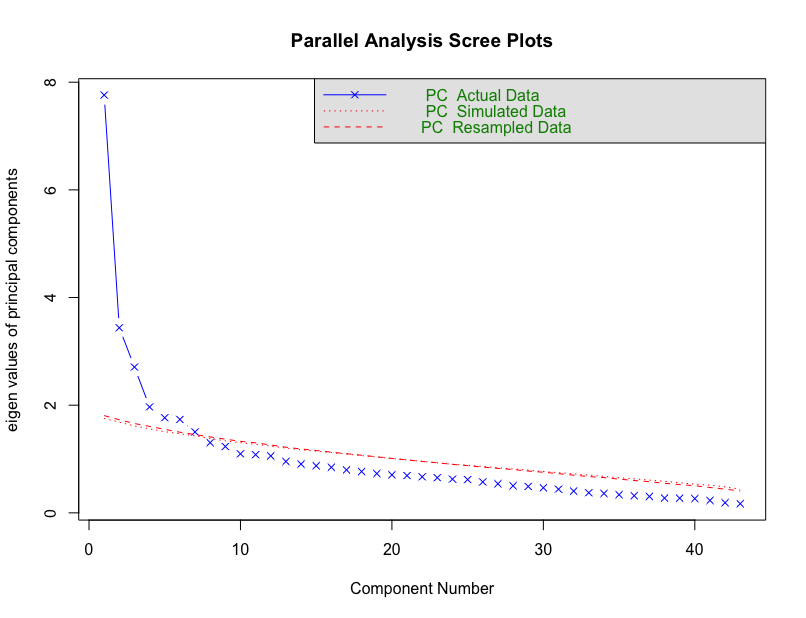


**Figure S2. Seven-Factor Structure of ASD-GIRBI (36 items)**


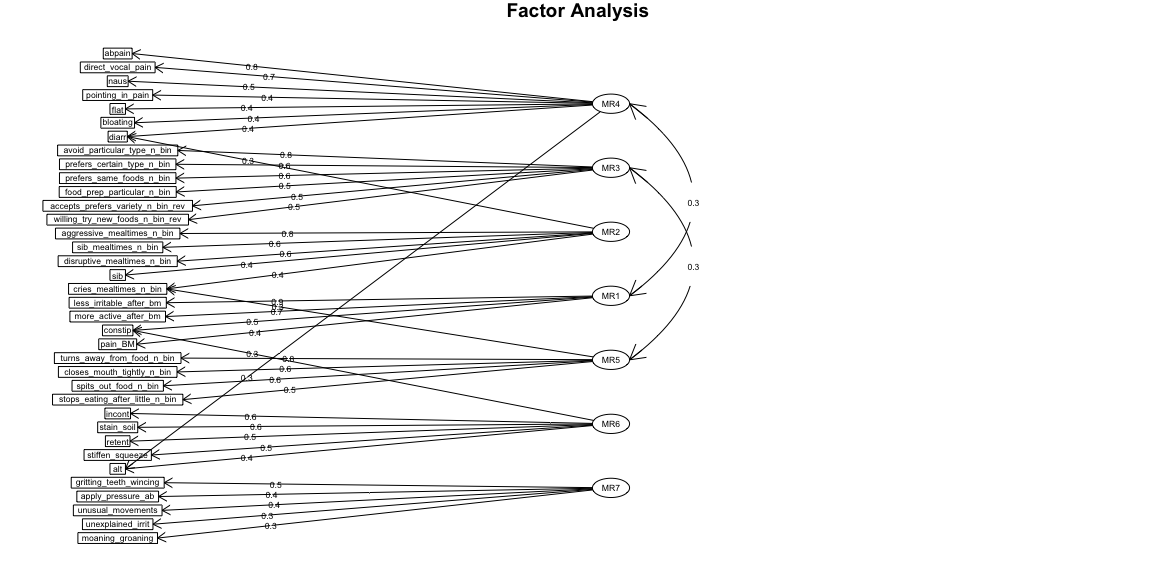

Supplement: Supplementary file 2 — Appendix S2: Supporting Information [file AUR-15-1142-s002.docx]
